# The Impact of Low Skeletal Muscle Mass on Short- and Long-Term Outcomes After Cytoreductive Surgery and Hyperthermic Intraperitoneal Chemotherapy

**DOI:** 10.1245/s10434-022-11941-2

**Published:** 2022-06-01

**Authors:** Michelle V. Dietz, Job P. van Kooten, Jeroen L. A. van Vugt, Alexandra R. M. Brandt-Kerkhof, Cornelis Verhoef, Eva V. E. Madsen

**Affiliations:** Department of Surgical Oncology, Erasmus MC Cancer Institute, Rotterdam, The Netherlands

## Abstract

**Background:**

Cytoreductive surgery (CRS) with hyperthermic intraperitoneal chemotherapy (HIPEC) is a potentially curative treatment for peritoneal metastases from colorectal cancer (CRC) or pseudomyxoma peritonei (PMP). Because of the considerable morbidity of this treatment, optimal patient selection is key. This study aimed to assess the impact of low skeletal muscle mass (SMM) on outcomes after CRS-HIPEC.

**Methods:**

Patients who underwent CRS-HIPEC between 2014 and 2020 at a tertiary center were included. SMM was measured on computed tomography by means of the L3 muscle index. Postoperative complications and survival outcomes were compared between groups by use of logistic regression and Kaplan-Meier survival analyses.

**Results:**

Of 284 included patients, 149 had low SMM. Occurrence of severe postoperative complications did not differ between groups (28.9% for patients with low vs. 34.1% for patients with normal SMM). Low SMM was not associated with postoperative complications (*p* = 0.344). For CRC patients, no significant differences were observed in disease-free (DFS) or overall survival (OS) between patients with low (median DFS 7 months [IQR 4–14], median OS 33 months [IQR 14–NR]) and patients with normal SMM (median DFS 8 months [IQR 5–20], median OS 35 months [IQR 18–NR]). Regarding PMP, survival outcomes did not significantly differ between groups (3-year DFS 47.3% for patients with low SMM vs. 54.5% for patients with normal SMM, *p* = 0.676; 3-year OS 70.8% vs. 90.9% respectively, *p* = 0.172).

**Conclusions:**

Low SMM could not be identified as a predictor of severe complications or survival outcomes after CRS-HIPEC.

**Supplementary Information:**

The online version contains supplementary material available at 10.1245/s10434-022-11941-2.

Cytoreductive surgery (CRS) combined with intraoperative hyperthermic intraperitoneal chemotherapy (HIPEC) is considered to be a potentially curative treatment for selected patients with peritoneal metastases (PM) from colorectal carcinoma (CRC) or pseudomyxoma peritonei (PMP). This extensive surgical treatment significantly improves the survival of CRC patients compared with systemic chemotherapy, resulting in 5-year survival rates of up to 40%.^1–5^ For PMP patients CRS-HIPEC is considered the golden standard with 5-year survival rates of 74%.^6^ Despite these improvements in survival, CRS-HIPEC is associated with considerable postoperative morbidity. Severe postoperative complications are reported in approximately 30% of the patients.^4, 7, 8^ Previous studies have shown an association between the occurrence of postoperative complications and impaired survival outcomes.^6, 9, 10^ Hence, for this select patient population, it is of great importance to identify risk factors for postoperative outcomes that could aid in preoperative patient selection.

With the increasing emphasis on prehabilitation in cancer surgery, potential risk factors associated with the nutritional status are widely investigated. As the number of obese cancer patients is rising, the utility of factors like BMI and weight loss is under debate.^11^ A potential risk factor of interest is sarcopenia, which is mainly determined by the loss of skeletal muscle mass (SMM) and can easily remain unnoticed in obese patients. Several studies showed that SMM was an independent predictor of outcomes after colorectal cancer surgery, and was better in predicting outcomes than other factors representing the patients’ nutritional status, like BMI or albumin.^11–16^ A few studies report on the impact of SMM in patients undergoing CRS-HIPEC for PM from CRC or PMP, and conflicting results have been published.^17–20^

The aim of this retrospective study was to identify the impact of low SMM on postoperative outcomes after CRS-HIPEC for these patients. The hypothesis was that low SMM can be a valid predictor of severe postoperative complications and impaired survival outcomes. Hence, preoperative SMM measurement can potentially aid in preoperative patient selection.

## Methods

### Study Population

All patients who underwent CRS-HIPEC for PM from CRC or PMP at the Erasmus MC Cancer Institute in Rotterdam, the Netherlands, between March 2014 and June 2020 were included in this study. Erasmus MC is a university hospital and tertiary referral center for patients with extensive (metastasized) colorectal cancer. Patients were excluded if a suitable preoperative computed tomography (CT) image or patient body height, both essential for SMM measurement, were not available. Relevant patient and disease-related characteristics, operation details, and postoperative outcomes were extracted from a prospectively maintained database. This study was approved by the local Medical Ethics Review Committee of Erasmus MC (MEC-2018-1286).

### Surgical Procedure

CRS-HIPEC procedures were performed by a specialized surgical team, in accordance with the Dutch CRS and HIPEC protocol.^21, 22^ After abdominal access via laparotomy, the peritoneal cancer index (PCI) according to Jacquet and Sugarbaker was used to estimate the tumor load.^23^ For patients with PM from CRC, cytoreductive surgery was performed if the PCI score was under 20 and/or the specialized surgeons presumed the PM to be resectable. For patients with PM from PMP, the PCI score was not considered for determining CRS-HIPEC feasibility.

### Postoperative Monitoring

Patients were postoperatively treated following standard local protocol for CRS-HIPEC. Postoperative complications were categorized according to the Clavien-Dindo classification.^24^ Severe postoperative complications were defined as Clavien-Dindo grade 3 or higher (i.e., re-intervention, extended ICU stay/readmission to ICU, or treatment-related death). In case of multiple complications, the highest-grade complication was registered. The postoperative period was defined as 30 days after CRS-HIPEC, or the duration of the entire hospital stay, when exceeding 30 days.

### Follow-Up

Follow-up was performed at the outpatient clinic by use of CT imaging and monitoring of the carcino-embryonal antigen (CEA). CEA was determined every 3 months and a CT examination was performed every 6 months during the first 2 postoperative years. After 2 years, the follow-up interval for CEA was 6 months and a CT scan was made every 12 months. A CT scan was also performed in case of increasing CEA levels or complaints, suspicious for recurrent disease. Follow-up was completed after a disease-free interval of 5 years following CRS-HIPEC.

### Skeletal Muscle Mass (SMM) Measurement

**Fig. 1:**
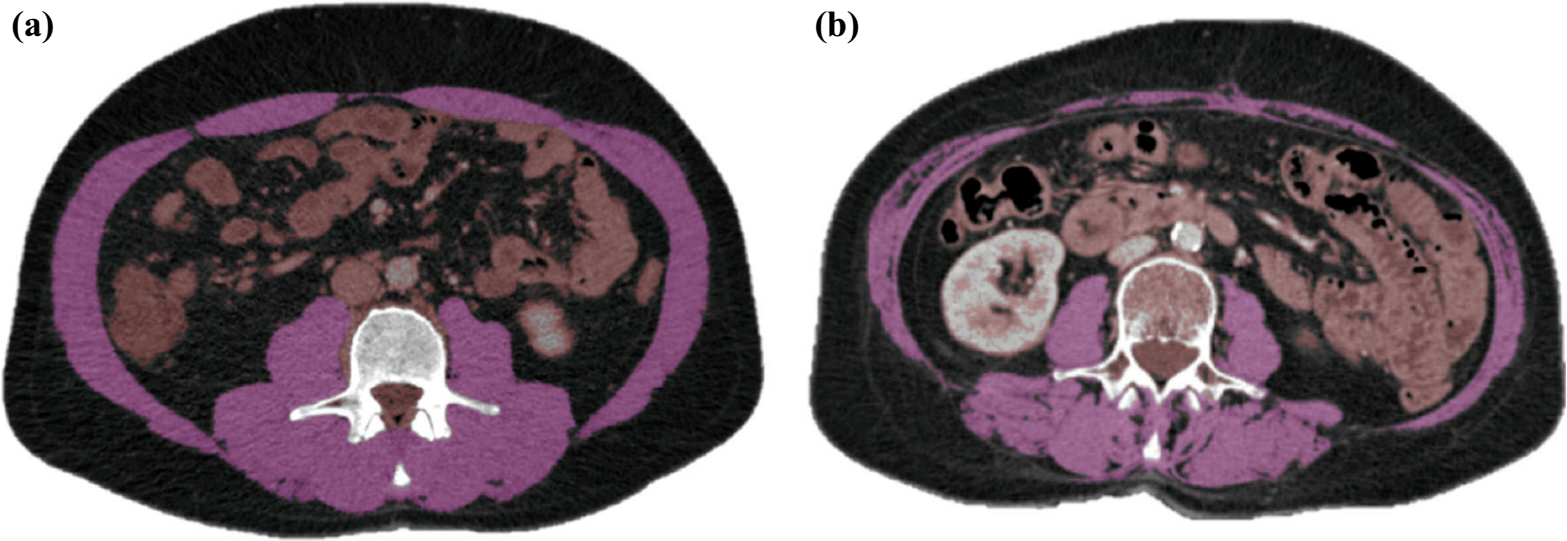
Axial CT slice at the level of the third lumbar vertebra of a male patient with normal SMM (**A**) and of a female patient with low SMM (**B**) with peritoneal metastasis from colorectal carcinoma. The psoas, paraspinal, transverse abdominal, external oblique, internal oblique, and rectus abdominis muscles are outlined in *purple* (threshold of − 30 to +150 HU)

Abdominal CT was routinely performed during preoperative assessment. For patients treated with neo-adjuvant chemotherapy, SMM measurements were performed on the CT scan that was made after neo-adjuvant treatment. SMM was determined by using FatSeg software (developed by the Biomedical Imaging Group Rotterdam of Erasmus MC, Rotterdam, the Netherlands, based on MeVisLab [Mevis Medical Solutions, Bremen, Germany]).^25^ In summary, SMM was measured twice at the level of the third lumbar vertebra (L3) on two different slices showing both transversal processes. The psoas, paraspinal, transverse abdominal, external oblique, internal oblique, and rectus abdominis muscles were manually traced (Fig. 1). The SMM area was computed automatically using the preset Hounsfield unit (HU) intensity thresholds (between –30 and +150), and was expressed in square centimeters. The L3 muscle index was calculated by dividing the average of the two L3 muscle area measurements by the squared patient height (cm^2^/m^2^). Low SMM was defined using the cut-off values of 43 cm^2^/m^2^ for men with BMI < 25 cm^2^/m^2^, 53 cm^2^/m^2^ for men with BMI ≥ 25 and 41 cm^2^/m^2^ for women, independent of BMI. These were developed in an oncological population to predict survival.^11^ The SMM measurements were performed by a member of the research group (MD). A second investigator (JK) performed a random control on 10% of the examinations.

### Primary and Secondary Outcomes

The primary outcome of this study was defined as the occurrence of severe postoperative complications (i.e., Clavien-Dindo grade 3 or higher). Secondary outcomes were disease-free survival (DFS) and overall survival (OS). DFS was defined as the time interval in months between CRS-HIPEC and date of recurrence or death. OS was defined as the time interval in months between CRS-HIPEC and date of death. Information on survival status was obtained from the national civil registry. When no event occurred, patients were censored at the date of the last follow-up visit for DFS or the date of last request of survival status for OS.

### Statistical Analysis

Continuous variables were expressed as median with interquartile range (IQR). Categorical variables were presented as counts with percentages. Continuous variables were compared between patients with low and normal SMM using the Mann-Whitney *U* test. Categorical variables were compared using the chi-square test or Fisher’s exact test if less than 5 events occurred. An intra-class correlation coefficient (ICC) based on a mean-rating (*k* = 3), absolute-agreement, 2-way mixed-effects model, was estimated to investigate the reliability of the SMM measurements of the two investigators. To determine the effect of low SMM on the occurrence of severe postoperative complications, corrected for other possible risk factors, multivariable linear regression with backward selection was used. Gender, age, BMI, ASA (American Society of Anesthesiologists) classification, and primary tumor type were entered in the multivariable model. Smoking history, PCI, presence of anastomosis and intraoperative blood loss are known predictive variables and were also entered in the model.^26–28^ The log-rank was used to compare OS and DFS between patients with low and normal SMM. Survival analysis was performed separately for CRC and PMP patients because of different prognoses. All tests were two-sided and differences were considered statistically significant when *p* < 0.05. All statistical analyses were performed using Statistical Package for Social Sciences (SPSS) version 25.0 (IBM Corporation, Armonk, NY, USA). Kaplan-Meier survival curves were created using R version 4.0.2 (https://www.r-project.org/).

## Results

During the study period, 244 patients (83.6%) underwent CRS-HIPEC for PM from CRC and 48 patients (16.4%) from PMP. Five CRC patients and three PMP patients were excluded from analyses because of the absence of a suitable preoperative CT for SMM measurement. The degree of reliability between the SMM measurements of the two investigators was high. The single measure ICC was 0.924 (95% CI 0.824–0.964, *p* < 0.001).

### Baseline and Intraoperative Characteristics

A total of 149 patients (52.5%) had low SMM. Baseline and intraoperative characteristics are displayed in Tables 1 and 2, respectively. Patients with low SMM were significantly more often woman than patients with normal SMM (55.0% vs. 42.2%, *p* = 0.031). Body mass index (BMI) was significantly lower for patients with low SMM (median 25.0 vs. 27.5, *p* < 0.001). Other baseline characteristics and intraoperative characteristics did not significantly differ between the groups. The median interval from preoperative CT to surgery was 5 weeks for both patients with low (IQR 3–8) and normal SMM (IQR 2–8).

**Table 1:** Baseline Characteristics

	Total	Low SMM	Normal SMM	*p*-value
	*N* = 284 (100)	*N* = 149 (52.5)	*N* = 135 (47.5)	
Gender				
Male	145 (51.1)	67 (45.0)	78 (57.8)	0.031*
Female	139 (48.9)	82 (55.0)	57 (42.2)	
Age (years)	62 [53–70]	63 [53–70]	61 [53–69]	0.593
BMI (kg/m^2^)	25.9 [23.1–29.2]	25.0 [22.0–27.4]	27.5 [24.3–30.5]	< 0.001*
Smoking (past or current)				
Yes	126 (46.2)	69 (49.6)	57 (42.5)	0.239
No	147 (53.8)	70 (50.4)	77 (57.5)	
Missing	11 (3.9)	10 (6.7)	1 (0.7)	
Diabetes				
Yes	31 (11.0)	19 (12.8)	12 (8.9)	0.288
No	252 (89.0)	129 (87.2)	123 (91.1)	
Missing	1 (0.4)	1 (0.7)	0 (0)	
Hypertension				
Yes	77 (27.4)	37 (25.2)	40 (29.9)	0.380
No	204 (72.6)	110 (74.8)	94 (70.1)	
Missing	3 (1.1)	2 (1.3)	1 (0.7)	
ASA-classification				
1	45 (16.1)	23 (15.8)	22 (16.5)	0.975
2	174 (62.4)	91 (62.3)	83 (62.4)	
≥ 3	60 (21.5)	32 (21.9)	28 (21.1)	
Missing	5 (1.8)	3 (2.0)	2 (1.5)	
Primary tumor				
PMP	45 (15.8)	24 (16.1)	21 (15.6)	0.467
Appendix	16 (5.6)	6 (4.0)	10 (7.4)	
CRC	223 (78.5)	119 (79.9)	104 (77.0)	
Primary location CRC				
Ascending colon	82 (36.8)	48 (40.3)	34 (32.7)	0.613
Transverse colon	19 (8.5)	9 (7.6)	10 (9.6)	
Descending colon	23 (10.3)	14 (11.8)	9 (8.7)	
Sigmoid	69 (30.9)	34 (28.6)	35 (33.7)	
Rectum	30 (13.5)	14 (11.8)	16 (15.4)	
T stage primary tumor^a^				
T1	6 (2.6)	2 (1.6)	4 (3.6)	0.651
T2	10 (4.3)	4 (3.3)	6 (5.4)	
T3	105 (44.9)	57 (46.7)	48 (42.9)	
T4	113 (48.3)	59 (48.4)	54 (48.2)	
Missing	5 (2.1)	3 (2.4)	2 (1.8)	
N stage primary tumor^a^				
N0	64 (28.4)	39 (32.8)	25 (23.6)	0.311
N1	88 (39.1)	44 (37.0)	44 (41.5)	
N2	73 (32.4)	36 (30.3)	37 (34.9)	
Missing	14 (5.9)	6 (4.8)	8 (7.0)	
M stage primary tumor^a^				
M0	100 (48.5)	51 (46.4)	49 (51.0)	0.503
M1	106 (51.5)	59 (53.6)	47 (49.0)	
Missing	33 (13.8)	15 (12.0)	18 (15.8)	
Liver metastases^a,b^				
Yes	23 (9.6)	16 (12.8)	7 (6.1)	0.081
Differentiation^a^				
Good	31 (16.0)	18 (17.0)	13 (14.8)	0.930
Moderate	129 (66.5)	71 (67.0)	58 (65.9)	
Poor	18 (9.3)	9 (8.5)	9 (10.2)	
Signet	16 (8.2)	8 (7.5)	8 (9.1)	
Missing	45 (18.8)	19 (15.2)	26 (22.8)	
Mucinous^a^				
Yes	50 (23.1)	21 (18.3)	29 (28.7)	0.069
No	166 (76.9)	94 (81.7)	72 (71.3)	
Missing	23 (9.6)	10 (8.0)	13 (11.4)	
Histopathology PMP^c^				
DPAM	39 (86.7)	21 (87.5)	18 (85.7)	0.860
PMCA	6 (13.3)	3 (12.5)	3 (14.3)	
PMCA-I	0 (0)	0 (0)	0 (0)	
PM onset^a^				
Synchronous	113(47.3)	65 (52.0)	48 (42.1)	0.126
Metachronous	126 (52.7)	60 (48.0)	66 (57.9)	
PSS				
0	25 (8.8)	12 (8.1)	13 (9.6)	0.925
1	47 (16.5)	25 (16.8)	22 (16.3)	
2	201 (70.8)	107 (71.8)	94 (69.6)	
3	11 (3.9)	5 (3.4)	6 (4.4)	
Neo-adjuvant chemotherapy^d^				
Yes	33 (11.6)	22 (14.8)	11 (8.1)	0.082
CT-to-surgery interval (weeks)	5 [2–8]	5 [3–8]	5 [2–8]	0.326

Continuous variables are shown as median [IQR]. Frequencies are shown as N (%), excluding ‘missing’

*BMI* body mass index, *ASA* American Association for Anesthesiology, *PMP* pseudomyxoma peritonei, *CRC* colorectal carcinoma, *PM* peritoneal metastasis, *DPAM* disseminated peritoneal adenomucinosis, *PMCA* peritoneal mucinous carcinomatosis, *PMCA-I* peritoneal mucinous carcinomatosis with intermediate features, *PSS* prior surgical score

^a^Proportion of CRC patients (*n* = 239)

^b^Synchronous liver metastasis to primary colorectal tumor

^c^Proportion of PMP patients (*n* = 45)

^d^Neo-adjuvant chemotherapy to CRS-HIPEC

*α < 0.05

**Table 2:** Intraoperative characteristics

	Total	Low SMM	Normal SMM	*p*-value
	*N* = 284 (100)	*N* = 149 (52.5)	*N* = 135 (47.5)	
PCI	11 [6–16]	10 [6–16]	12 [7–17]	0.156
CCR-score				
R1	277 (97.5)	147 (98.7)	130 (96.3)	0.340
R2a	5 (1.8)	1 (0.7)	4 (3.0)	
R2b	2 (0.7)	1 (0.7)	1 (0.7)	
Procedure time (min)	373 [304–438]	380 [306–435]	365 [303–451]	0.880
Blood loss (l)	1.0 [0.6–1.6]	0.9 [0.6–1.5]	1.0 [0.6–1.8]	0.265
HIPEC regimen				
MMC	261 (91.9)	140 (94.0)	121 (89.6)	0.182
Oxaliplatin	23 (8.1)	9 (6.0)	14 (10.4)	
Resections				
Omentum	267 (94.0)	142 (95.3)	125 (92.6)	0.336
Peritoneum	214 (75.4)	116 (77.9)	98 (72.6)	0.304
Diaphragm	60 (21.1)	28 (18.8)	32 (23.7)	0.311
Stomach	4 (1.4)	3 (2.0)	1 (0.7)	0.363
Small bowel	75 (26.4)	41 (27.5)	34 (25.2)	0.656
Colon	169 (59.5)	82 (55.0)	87 (64.4)	0.107
Rectum	60 (21.1)	31 (20.8)	29 (21.5)	0.889
Gallbladder	18 (6.3)	10 (7.4)	8 (5.4)	0.481
Pancreas	12 (4.2)	6 (4.4)	6 (4.0)	0.861
Spleen	22 (7.7)	8 (5.9)	14 (9.4)	0.275
Pelvic organs^a^	160 (56.3)	89 (59.7)	71 (52.6)	0.226
Synchronous liver treatment^b^	33 (11.6)	22 (14.8)	11 (8.1)	0.082
Anastomosis				
Yes	163 (57.4)	81 (54.4)	82 (60.7)	0.278
Median number/patient	1 [0–1]	1 [0–1]	1 [0–1]	0.723
Stoma				
Ileostomy	15 (5.3)	7 (4.7)	8 (5.9)	0.106
Colostomy	85 (29.9)	37 (24.8)	48 (35.6)	

Continuous variables are shown as median [IQR]. Frequencies are shown as *N* (%), excluding ‘missing’

*PCI* Peritoneal Cancer Index, *CCR* completeness of cytoreduction, *MMC* mitomycin-C

^a^Pelvic organs including urinary bladder, ovaries, uterus, ureters and pelvis

^b^Liver treatment during CRS-HIPEC procedure: hepatic resection (*n* = 24) or radiofrequency ablation (RFA, *n* = 9)

### Postoperative Outcomes

There were no differences in the occurrence of severe postoperative complications in general (i.e., Clavien-Dindo 3 or higher) between the groups (Table 3). Patients with normal SMM had a perforation significantly more often (i.e., bowel perforation *n* = 6, gallbladder perforation *n* = 1) than patients with low SMM (bowel perforation *n* = 1, *p* = 0.022). Other postoperative outcomes did not significantly differ between the groups. Low SMM was not associated with severe postoperative complications in univariate logistic regression analysis (OR 0.79, 95% CI 0.48–1.30, *p* = 0.344; Table 4). Significant risk factors in univariate analysis were male gender (OR 2.67, 95% CI 1.58–4.53, *p* < 0.001), smoking (OR 1.76, 95% CI 1.06–2.94, *p* = 0.030) and more intraoperative blood loss (OR 1.34, 95% CI 1.04–1.73, *p* = 0.021). In a multivariable analysis, male gender (OR 2.67, 95% CI 1.58–4.53, *p* < 0.001), smoking (OR 1.76, 95% CI 1.06–2.94, *p* = 0.030), and more intraoperative blood loss (OR 1.34, 95% CI 1.04–1.73, *p* = 0.021) remained significantly associated with the occurrence of severe postoperative complications.

**Table 3:** Postoperative outcomes

	Total	Low SMM	Normal SMM	*p*-value
	*N* = 284 (100)	*N* = 149 (52.5)	*N* = 135 (47.5)	
Length of stay (days)	16 [12–20]	16 [12–19]	16 [12–22]	0.594
Complications (any grade)				
Any complication	181 (63.7)	98 (65.8)	83 (61.5)	0.453
Anastomotic leakage^a^	26 (9.2)	13 (8.7)	13 (9.6)	0.792
Perforation^b^	8 (2.8)	1 (0.7)	7 (5.2)	0.022*
Postoperative hemorrhage	11 (3.9)	8 (5.4)	3 (2.2)	0.170
Intra-abdominal abscess	33 (11.6)	16 (10.7)	17 (12.6)	0.626
Ileus/gastroparesis^c^	48 (16.9)	28 (18.8)	20 (14.8)	0.372
Wound infection	20 (7.0)	7 (4.7)	13 (9.6)	0.105
Wound dehiscence	8 (2.8)	2 (1.3)	6 (4.4)	0.115
Chylous leakage	10 (3.5)	5 (3.4)	5 (3.7)	0.874
Pneumonia	15 (5.3)	10 (6.7)	5 (3.7)	0.258
Pulmonary embolism	9 (3.2)	3 (2.0)	6 (4.4)	0.243
Cardiac complications	13 (4.6)	5 (3.4)	8 (5.9)	0.301
UTI	20 (7.0)	13 (8.7)	7 (5.2)	0.244
Severe complication^d^	89 (31.3)	43 (28.9)	46 (34.1)	0.344
Reoperations	43 (15.1)	20 (13.4)	23 (17.0)	0.396
Clavien-Dindo grade				
I	19 (6.7)	11 (7.4)	8 (5.9)	0.946
II	73 (25.7)	42 (28.2)	31 (23.0)	
IIIa	39 (13.7)	19 (12.8)	20 (14.8)	
IIIb	32 (11.3)	16 (10.7)	16 (11.9)	
IVa	10 (3.5)	5 (3.4)	5 (3.7)	
IVb	2 (0.7)	1 (0.7)	1 (0.7)	
V	6 (2.1)	2 (1.3)	4 (3.0)	
Adjuvant chemotherapy				
Yes	38 (13.4)	20 (13.4)	18 (13.3)	0.982

Continuous variables are shown as median [IQR]. Frequencies are shown as *N* (%), excluding ‘missing’

*UTI* urinary tract infection

^a^Proportion of patients with a bowel anastomosis after CRS-HIPEC (*n* = 163)

^b^Perforation: bowel perforation (*n* = 7), gallbladder perforation (*n* = 1)

^c^Ileus (*n* = 11), gastroparesis (*n* = 42)

^d^Clavien-Dindo classification ≥ III (i.e., re-intervention, extended ICU stay/readmission to ICU, or treatment-related death)

*α < 0.05

**Table 4:** Logistic regression for predictors of severe postoperative complications (i.e., Clavien-Dindo ≥ 3)

	Univariable OR (95% CI)	*p*-value	Multivariable OR (95% CI)	*p*-value
Low SMM	0.79 (0.48–1.30)	0.344		
Gender				
Female	1		1	
Male	2.67 (1.58–4.53)	<0.001*	2.54 (1.44–4.48)	0.001*
Age (years)	1.02 (0.99–1.04)	0.199		
BMI (kg/m^2^)	1.01 (0.96–1.07)	0.648		
Smoking (past or current)	1.76 (1.06–2.94)	0.030*	2.19 (1.24–3.84)	0.007*
ASA-classification				
1	1			
2	1.14 (0.55–2.34)	0.725		
≥ 3	1.23 (0.53–2.85)	0.628		
Primary tumor				
CRC	1			
Appendix^a^	1.43 (0.50–4.09)	0.508		
PMP	1.44 (0.74–2.82)	0.281		
PCI	1.03 (0.99–1.06)	0.061		
Blood loss (l)	1.34 (1.04–1.73)	0.021*	1.46 (1.11–1.94)	0.008*
Anastomosis				
Yes	1.40 (0.83–2.33)	0.204		

*BMI* body mass index, *ASA* American Association of Anesthesiology, *PMP* pseudomyxoma peritonei, *CRC* colorectal carcinoma, *PCI* peritoneal cancer index

*α < 0.05

^a^Appendiceal adenocarcinoma

### Survival Outcomes

**Fig. 2:**
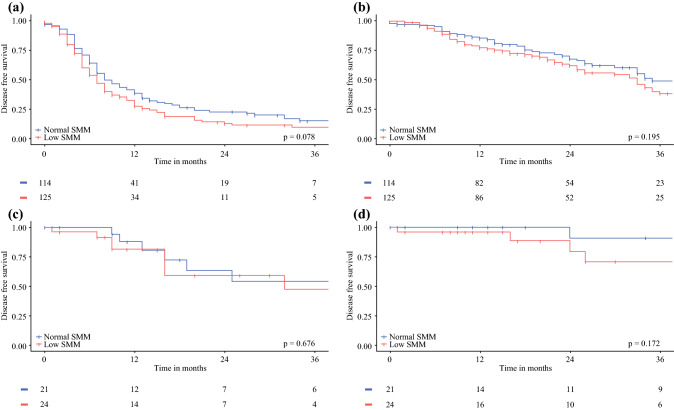
Kaplan-Meier survival curves for disease-free survival and overall survival for CRC (**a**, **b**) and PMP (**c**, **d**) patients with low versus normal SMM. The numbers at risk are displayed in the Tables *below* each graph. The log-rank *p*-values are displayed in the *bottom right corner* of each graph

Median follow-up time for surviving CRC patients was 24 months (IQR 12–37). A total of 179 CRC patients (73.4%) had recurrence of disease during follow-up and the median DFS was 8 months (IQR 4–16). There was no significant difference in median DFS between patients with low and patients with normal SMM (7 [IQR 4–14] vs. 8 months [IQR 5–20], *p* = 0.078; Fig. 2A). Median OS for CRC patients was 33 months (IQR 17–NR [not reached]). The median OS (33 months, IQR 14–NR) for CRC patients with low SMM did not significantly differ from CRC patients with normal SMM (35 months, IQR 18–NR, *p* = 0.195, Fig. 2B).

Median follow-up time for surviving PMP patients was 18 months (IQR 9–50). A total of 13 PMP patients (27.1%) had recurrence of disease during follow-up. For PMP patients the median DFS and OS were not yet reached. The 1- and 3-year DFS rates were 84.2% and 51.8%, respectively, for all PMP patients. For patients with low SMM, the 1- and 3-year DFS rates were 81.3% and 47.3%, respectively, compared with 87.8% and 54.5% for patients with normal SMM (*p* = 0.676, Fig. 2C). The 1- and 3- year OS rates were 97.7% and 80.4%, respectively, for all PMP patients. The 1- and 3-year OS rates were 95.8% and 70.8%, respectively, for patients with low versus 100% and 90.9% for patients with normal SMM (*p* = 0.172, Fig. 2D).

### Additional Analysis of Patients Who Did Not Receive Neo-adjuvant Chemotherapy to CRS-HIPEC

There was no significant difference in the occurrence of severe postoperative complications in general between groups for patients that did not receive neo-adjuvant chemotherapy to CRS-HIPEC (32.2% for patients with normal SMM vs. 29.9% for patients with low SMM, *p* = 0.689). In a multivariable analysis, male gender (OR 2.75, 95% CI 1.49–5.06, *p* = 0.001), smoking (OR 2.19, 95% CI 1.23–4.13, *p* = 0.008), and more intraoperative blood loss (OR 1.45, 95% CI 1.08–1.93, *p* = 0.013) were significantly associated with the occurrence of severe postoperative complications. For CRC patients that did not receive neo-adjuvant chemotherapy to CRS-HIPEC, median DFS was significantly shorter for patients with low SMM (7 [IQR 4–13] vs. 8 months [IQR 5–20], *p* = 0.019; Supplementary Fig. 1A). Median OS was 35 months for the normal SMM group [IQR 19–NR] versus 33 months in the low SMM group [IQR 15–56] (*p* = 0.124, Supplementary Fig. 1B). None of the PMP patients received neo-adjuvant chemotherapy to CRS-HIPEC.

## Discussion

The aim of this study was to identify the impact of low skeletal muscle mass (SMM) on postoperative outcomes in patients undergoing CRS-HIPEC for peritoneal metastases (PM) from colorectal carcinoma (CRC) or pseudomyxoma peritonei (PMP). The current study found no association between low SMM and the occurrence of severe postoperative complications or survival outcomes.

Previous studies, reporting on the impact of sarcopenia in general colorectal cancer surgery, showed that low SMM was associated with higher rates of postoperative complications and impaired survival.^12, 16, 29, 30^ Therefore, low SMM has been proposed to aid in preoperative patient selection. This could be especially helpful for patients suffering from PM from CRC, because of their limited prognosis and considerable postoperative morbidity after CRS-HIPEC. CT scans are routinely performed as part of the preoperative assessment for CRS-HIPEC. Measurement of SMM on these CT scans could be used in preoperative patient selection. However, the current study could not reproduce an association between low SMM and postoperative outcomes in patients undergoing CRS-HIPEC. This is in line with other studies that investigated the impact of SMM in this specific patient population.^18–20^ An explanation for the discrepancy between general colorectal surgery and CRS-HIPEC might be the strict preoperative patient selection for CRS-HIPEC, mainly based on fitness for major surgery, leading to (strong) selection bias. Indeed, the vast majority of patients (around 80%) had an ASA classification of 1 or 2. The impact of low SMM on postoperative outcomes might be smaller in this strictly selected population, in contrast to the less selected patient population undergoing general colorectal cancer surgery.

One study regarding postoperative complications in patients undergoing CRS-HIPEC for PM from CRC, by Van Vugt et al., reported that patients with skeletal muscle depletion had significantly more reoperations than patients with normal SMM. They also found that lower SMM as a continuous measure, was independently associated with a higher rate of severe postoperative complications.^17^ However, they did not find this association when using SMM as a dichotomous variable (i.e., low vs. normal SMM). To increase the potential for clinical use, the current authors decided to use the SMM as a dichotomous variable. However, there is no consensus in the field as to which cut-off values should be used to define low SMM. Previous studies, including the study by Van Vugt et al., used cut-off values defined by Prado et al.^31^ These values have been acknowledged in an international consensus statement on cancer cachexia and have been validated for mortality prediction in obese patients with pulmonary and gastrointestinal cancer.^32^ It is questionable whether these values are applicable in the current, mostly non-obese, cohort. Martin et al. more recently proposed cut-off values based on a general patient population with pulmonary and gastrointestinal malignancies, which were stratified for sex and BMI.^11^ These cut-offs were applied because they were considered more appropriate for the current study. Nevertheless, we could not demonstrate a relation between low SMM and severe postoperative complications or reoperations after CRS-HIPEC. Van Vugt et al. included patients that underwent CRS-HIPEC between 2005 and 2013, whereas the current cohort consists of patients that underwent CRS-HIPEC from 2014. Preoperative patient selection regarding CRS-HIPEC has most likely improved during the last decade, resulting in stricter patient selection.

Another explanation could be that patients with low SMM were significantly more often women. This is in line with the study of Martin et al., in which these cut-off values were proposed.^11^ In the current cohort, male gender was independently associated with severe postoperative complications. However, in multivariate analysis including sex, low SMM remained unassociated with severe postoperative complications. Besides one previous study that reported on a proportion of the current study population, the association between sex and postoperative complications has not been previously described for CRS-HIPEC.^33^ Previous studies on colorectal surgery reported an association between male sex and increased risk of anastomotic leakage.^34, 35^ One of these studies reported that the leak rate was especially high in men with low cancers.^35^ This study proposed that this might be explained by anatomical differences in the narrower male pelvis and hormonal differences that might influence the intestinal microcirculation. In the current cohort, other factors that were associated with severe postoperative complications were smoking and intraoperative blood loss. This conforms with previous studies on complications after CRS-HIPEC.^26, 27^

The cut-off values that were proposed by Martin et al. to classify SMM as low or normal, were based on SMM measurements on CT scans that were made before receiving any treatment for the measurement of SMM.^11^ In the current study, SMM measurements were performed on the most recent CT scan that was made for CRS-HIPEC. For patients that were treated with neo-adjuvant chemotherapy for CRS-HIPEC, this was the CT scan that was made after neo-adjuvant treatment. Previous studies have shown that neo-adjuvant therapy was associated with the loss of SMM.^36–38^ The post-therapy CT scan provides a more reliable view of the patients’ physical status at time of surgery. However, concerning the utility of SMM measurements in patient selection for CRS-HIPEC, the possible effect of neo-adjuvant chemotherapy on SMM should be taken into account. In the current cohort, only a minority of patients (12%) received neo-adjuvant chemotherapy, and there was no significant difference between groups. An additional analysis, excluding the patients that were treated with neo-adjuvant chemotherapy to CRS-HIPEC, showed similar outcomes regarding the occurrence of severe postoperative complications and OS. CRC patients with low SMM who did not receive neo-adjuvant chemotherapy had a significantly shorter DFS (7 months) than patients with normal SMM (8 months). Although not statistically significant, slightly more patients in the low SMM group received neo-adjuvant chemotherapy (14.8%) than in the normal SMM group (8.1%). This supports the hypothesis that neo-adjuvant chemotherapy is associated with the loss of SMM. Besides the significant difference in DFS in this subgroup analysis, there is a trend towards a slightly better DFS and OS for patients with normal SMM in the general study population. Low SMM might affect survival outcomes, but larger numbers of patients may be needed to support this hypothesis due to the highly selected population of patients undergoing CRS-HIPEC.

Regarding PMP, a study by Galan et al. showed that OS was significantly higher in patients with normal SMM.^20^ This difference was found in the first months after CRS-HIPEC, without a significant difference in the occurrence of severe postoperative complications. Galan et al. stated that patients with low SMM might have a higher risk of death when major complications occur. In the current cohort, the postoperative mortality for PMP patients was very low (i.e., *n* = 1), which explains why this difference in OS was not found.

The current study had some limitations. Due to the retrospective nature of the current study, additional data on muscle function or nutritional status could not be obtained. The European Working Group on Sarcopenia in Older People (EWGSOP) recently updated the European consensus on the definition and diagnosis of sarcopenia.^39, 40^ Whereas the EWGSOP previously recommended using the presence of both low muscle mass and low muscle function for the diagnosis of sarcopenia, the new consensus uses low muscle strength as the primary element of sarcopenia. Several studies proposed that muscle function (defined by factors like hand grip strength or cardiopulmonary exercise testing) might be better in reflecting a patients’ physical function or nutritional status than skeletal muscle mass.^41–44^ As skeletal muscle mass can be measured on routinely performed CT-scans, it is an easily available measure of the patients’ physical status. A more comprehensive picture of the patients’ physical and nutritional status might contribute to the prediction of postoperative outcomes in cancer patients. However, as mentioned previously, the patients undergoing CRS-HIPEC consist of a selected patient population. It is questionable whether a more comprehensive picture of the patients’ physical function provides significant additional value to patient selection for this specific patient population. Another limitation of this study was the limited follow-up period for surviving patients. Therefore, we presented 3-year survival data. For PMP patients, the sample size was small and median DFS and OS were not reached. Therefore, statements on the impact of low SMM on survival in these patients could not be made. Lastly, this study only investigated SMM as a predictor of postoperative outcomes. Sarcopenia, also defined by muscle weakness, might be a better predictor of postoperative outcomes. Previous studies reporting on the impact of sarcopenia on postoperative outcomes after general colorectal cancer surgery and CRS-HIPEC are inconsistent in the definition of sarcopenia. Future studies should investigate whether sarcopenia, defined by low SMM and muscle weakness, is a valid predictor of postoperative outcomes after CRS-HIPEC. In addition, other factors, such as weight loss and nutritional depletion, might also be relevant for the prediction of frailty and, consequently, of postoperative outcomes.

## Conclusions

This study showed that low SMM was not a predictor of postoperative outcomes after CRS-HIPEC. This is probably explained by strict patient selection, based on factors like fitness for major surgery. Morbidity after CRS-HIPEC is considerable, nonetheless. This morbidity might be more acceptable in patients with long-term disease-free and overall survival. Hence, future research should focus on the identification of prognostic factors useful in preoperative patient selection.

## Supplementary Information

Below is the link to the electronic supplementary material.

Supplementary Figure 1. Kaplan-Meier survival curves for disease-free survival (A) and overall survival (B) for CRC patients with low versus normal SMM. The numbers at risk are displayed in the table below the graphs. The Log rank p-values are displayed in the bottom right corner (TIF 35 KB)

## Race/Ethnicity Data

The race/ethnicity distribution was not reported, as this data was not collected in the database that was analyzed for this study. As this is a retrospective study, this data could not be obtained.
