# Supplementary figures and images for: The Impact of Low Skeletal Muscle Mass on Short- and Long-Term Outcomes After Cytoreductive Surgery and Hyperthermic Intraperitoneal Chemotherapy
Source: Ann Surg Oncol. 2022 Jun 1;29(9):5830–41. doi: 10.1245/s10434-022-11941-2 (PMC9356922; doi:10.1245/s10434-022-11941-2)

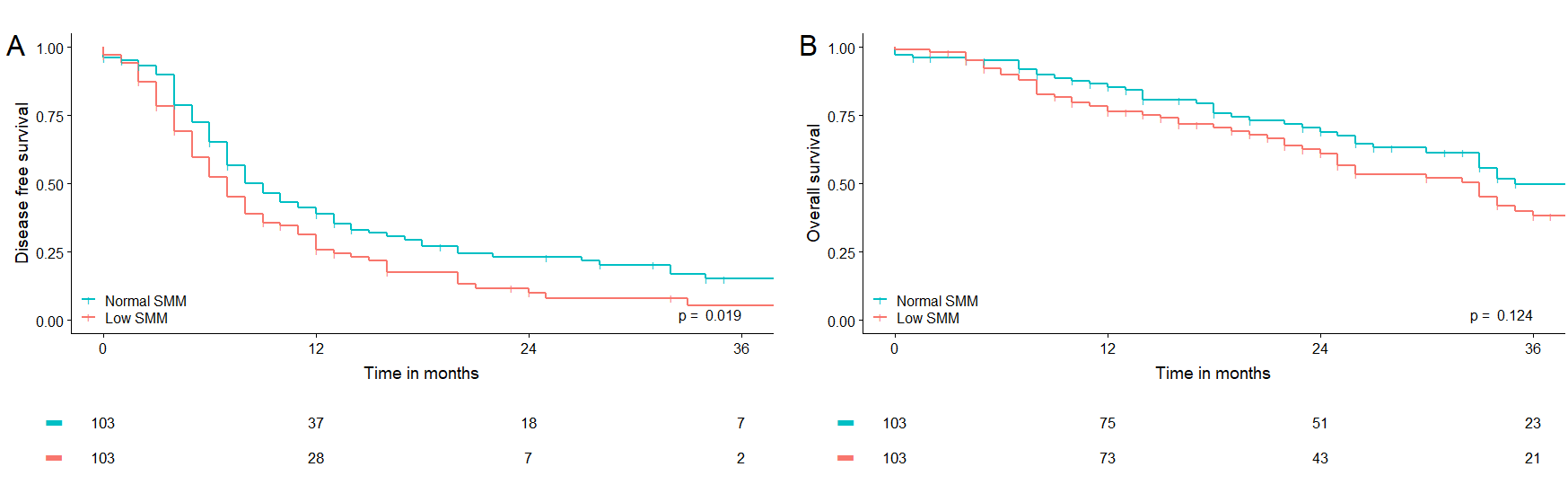

Supplement: Supplementary file 1 — Supplementary Figure 1. Kaplan-Meier survival curves for disease-free survival (A) and overall survival (B) for CRC patients with low versus normal SMM. The numbers at risk are displayed in the table below the graphs. The Log rank p-values are displayed in the bottom right corner (TIF 35 KB) [file 10434_2022_11941_MOESM1_ESM.tif]
